# Commentary: Reconstruction of Pharyngolaryngeal Defects with the Ileocolon Free Flap: A Comprehensive Review and How to Optimize Outcomes

**DOI:** 10.1055/s-0043-1777252

**Published:** 2024-02-22

**Authors:** Joseph M. Escandón, Eric Santamaría, Peter A. Prieto, Daniela Duarte-Bateman, Pedro Ciudad, Megan Pencek, Howard N. Langstein, Hung-Chi Chen, Oscar J. Manrique

**Affiliations:** 1 Division of Plastic and Reconstructive Surgery, Strong Memorial Hospital, University of Rochester Medical Center, Rochester, New York; 2 Department of Plastic and Reconstructive Surgery, Hospital General Dr. Manuel Gea Gonzalez, National Cancer Institute, Mexico City, Mexico; 3 Surgery Department, University of Rochester Medical Center, New York; Wilmot Cancer Institute, University of Rochester Medical Center, New York; 4 Division of Plastic and Reconstructive Surgery, Cleveland Clinic, Ohio; 5 Department of Plastic, Reconstructive and Burn Surgery, Arzobispo Loayza National Hospital, Lima, Peru; 6 Department of Plastic Surgery, China Medical University Hospital, Taichung, Taiwan

Reconstruction of Pharyngolaryngeal Defects with the Ileocolon Free Flap: A Comprehensive Review and How to Optimize Outcomes

Several reconstructive methods have been reported to restore the continuity of the aerodigestive tract following resection of pharyngeal and hypopharyngeal cancers. However, high complication rates have been reported after voice prosthesis insertion. In this setting, the ileocolon free flap (ICFF) offers a tubularized flap for reconstruction of the hypopharynx while providing a natural phonation tube. Herein, we systematically reviewed the current evidence on the use of the ICFF for reconstruction of the aerodigestive tract. A systematic literature search was conducted across PubMed MEDLINE, Web of Science, ScienceDirect, Scopus, and Ovid MEDLINE(R). Data on the technical considerations and surgical and functional outcomes were extracted. Twenty-one studies were included. The mean age and follow-up were 54.65 years and 24.72 months, respectively. An isoperistaltic or antiperistaltic standard ICFF, patch flap, or chimeric seromuscular-ICFF can be used depending on the patients' needs. The seromuscular chimeric flap is useful to augment the closure of the distal anastomotic site. The maximum phonation time, frequency, and sound pressure level (dB) were higher with ileal segments of 7 to 15 cm. The incidence of postoperative leakage ranged from 0 to 13.3%, and the majority was occurring at the coloesophageal junction. The revision rate of the microanastomosis ranged from 0 to 16.6%. The ICFF provides a reliable and versatile alternative for reconstruction of middle-size defects of the aerodigestive tract. Its three-dimensional configuration and functional anatomy encourage early speech and deglutition without a prosthetic valve and minimal donor-site morbidity.



